# Genetic Interaction of *APOE* and *FGF1* is Associated with Memory Impairment and Hippocampal Atrophy in Alzheimer’s Disease

**DOI:** 10.14336/AD.2018.0606

**Published:** 2019-06-01

**Authors:** Ya-Ting Chang, Hiroaki Kazui, Manabu Ikeda, Chi-Wei Huang, Shu-Hua Huang, Shih-Wei Hsu, Wen-Neng Chang, Chiung-Chih Chang

**Affiliations:** ^1^Department of Neurology, Kaohsiung Chang Gung Memorial Hospital, Chang Gung University College of Medicine, Kaohsiung 83301, Taiwan; ^2^Department of Psychiatry, Osaka University Graduate School of Medicine, Suita, Osaka 565-0871, Japan; ^3^Department of Nuclear Medicine, Kaohsiung Chang Gung Memorial Hospital, Chang Gung University College of Medicine, Kaohsiung 83301, Taiwan; ^4^Department of Radiology, Kaohsiung Chang Gung Memorial Hospital, Chang Gung University College of Medicine, Kaohsiung 83301, Taiwan

**Keywords:** APOE, episodic memory, FGF1, genetic interaction, hippocampus

## Abstract

The *APOE* and fibroblast growth factor 1 (*FGF1*) have both been associated with amyloid β accumulation and neurodegeneration. Investigation the effect of *APOE-FGF1* interactions on episodic memory (EM) deficits and hippocampus atrophy (HA) might elucidate the complex clinical-pathological relationship in Alzheimer’s disease (AD). EM performance and hippocampal volume (HV) were characterized in patients with mild AD based on *APOE*-ε4 carrier status (*APOE*-ε4 carriers versus non-carriers) and *FGF1* single nucleotide polymorphism (*FGF1-*rs34011-GG versus *FGF1-*rs34011-A-allele carriers). The clinical-pathological relationships within each genotypic group (ε4+/GG-carrier, ε4+/A-allele-carrier, ε4-/GG-carrier and ε4-/A-allele-carrier) were analyzed. There were no significant differences between the *FGF1-*rs34011-GG and *FGF1-*rs34011-A-allele carriers for the level of EM performance or HV (*p*> 0.05). The bilateral HV was significantly smaller and EM impairment was significantly worse in ε4+/GG-carrier than in ε4-/A-allele-carrier, and an interaction effect of *APOE* (*APOE*-ε4 carriers versus non-carriers) with *FGF1* (*FGF1-*rs34011-GG versus *FGF1-*rs34011-A-allele carriers) predicted EM impairment (F4,92= 3.516, p= 0.018) and structural changes in voxel-based morphometry. Our data shows that concurrent consideration of *APOE* and *FGF1* polymorphisms might be required to understand the clinical-pathological relationship in AD.

Sporadic Alzheimer’s disease (AD) is 70% heritable [1], and many genetic variants have been shown to influence the disease presentation and course [2]. Several single nucleotide polymorphisms (SNPs) have been identified to confer risk for AD using a genome-wide association approach [3-5]. Clinical-pathologically, presence of the apolipoprotein E (*APOE*)-ε4 allele is the most well-known genetic factor that leads to hippocampal atrophy (HA) [6-9], which is crucial in encoding and retrieving new information [10, 11]. The detrimental effect of the *APOE*-ε4 allele on the hippocampus is believed to be related to episodic memory (EM) deficits [12, 13], a characteristic of AD [10]. However, the *APOE*-ε4 carrier status has been shown inconsistent impact on EM performance [14], with both an adverse effect of *APOE*-ε4 allele on EM performance [15] and no significant EM deficits in the *APOE*-ε4 carriers [8]. The pathogenesis behind the inconsistent relationship is not fully understood.

Fibroblast growth factor 1 (*FGF1*) is a potent mitogen and is involved in cell survival [16]. Of relevance to neurodegeneration in AD, *FGF1* appears to be involved in the calcium homeostasis [17, 18] and expression of N-methyl-D-aspartate receptor [19] to protect vulnerable neurons in the hippocampus and entorhinal cortices against excito-toxicity. Moreover, *FGF1* has been shown to facilitate the gathering of reactive astrocytes around AD-related plaques in the regions susceptible to Aβ plaques [20]. Several SNPs in *FGF1* are identified, of which the *FGF1* promoter rs34011 (-1385G/A) SNP has been shown to be related to several pathologies via its function in controlling *FGF1* [21, 22]. The rs34011-A-allele genotype of *FGF1* has been associated with a lower AD risk than rs34011-GG genotype [22], although the results have not been consistent [23].

Biophysically, the *APOE* has been shown to modulate Aβ accumulation [24] and regulate apoE production, which is involved in neuronal regeneration in the hippocampus [25, 26]. In this regard, *FGF1* also plays an important role in the AD-related pathologic process of neurodegeneration [17-19] and Aβ deposition [20]. Further studies are needed to understand whether *APOE*-*FGF1* interactions are phenotypic relevant and contribute to the clinical and pathological heterogeneity of AD [26, 27].

In the present study, we compared the pattern of EM performance and HA in 97 patients with AD harboring various *APOE*-*FGF1* genetic variations. We hypothesized that an interaction effect of *APOE* (*APOE*-ε4 carriers versus non-carriers) with *FGF1* (rs34011-GG versus rs34011-A-allele carriers) predicted HA and EM deficits. We investigated whether the *FGF1* (rs34011) genotype modulates HA and EM deficits in *APOE*-ε4 carriers. Through these analyses, we aimed to explore the contribution of these genetic variants to AD-associated pathologic processes.

## MATERIALS AND METHODS

### Inclusion and Exclusion Criteria

Ninety-seven patients with AD were enrolled from the Department of Neurology of Chang Gung Memorial Hospital from 2011 to 2017. The patients were included on the basis of consensus of panels composed of neurologists, neuropsychologists, neuroradiologists, and experts in nuclear medicine. AD was diagnosed according to the International Working Group criteria [28] with a clinical diagnosis of typical AD. All of the AD patients were under stable treatment with acetylcholine esterase inhibitors from the time of diagnosis. Only the patients with mild-stage AD with a Clinical Dementia Rating (CDR) score of 0.5 or 1 were included. The exclusion criteria were a history of clinical stroke, a modified Hachinski ischemic score> 4 [29], and depression.

### Study Design

The study was approved by Chang Gung Memorial Hospital’s Institutional Review Committee on Human Research, and all of the participants and their authorized caregivers provided written informed consent. Cognitive testing and magnetic resonance imaging (MRI) were all performed within a period of 4 weeks.

### Genotyping

Genomic DNA was extracted from blood samples using a commercial kit (Qiagen, Gentra Puregene Blood Kit), followed by genotyping for G-1385A SNP at the *FGF1* gene using the polymerase chain reaction-restriction fragment length polymorphism method [22]. The *APOE* genotype was also determined [30]. Genotyping was conducted with the operator blinded to the clinical data. The patients were classified into two genotypic groups based on the *FGF1* SNP: rs34011-GG carriers (GG-carriers) and rs34011-A-allele carriers (A-allele-carriers). Those with one or two *APOE*-ε4 alleles were defined as *APOE*-ε4 carriers (ε4+ carriers) [30] and the others as *APOE*-ε4 non-carriers (ε4- carriers). Among the 38 ε4+ carriers, 33 carriers were heterozygous (ε3/ε4) and five carriers were homozygous (ε4/ε4), whereas 55 ε4 non-carriers were homozygous (ε3/ε3), three ε4 non-carriers were heterozygous (ε2/ε3), and only one ε4 non-carriers were homozygous (ε2/ε2). In the meanwhile, 12 patients were *FGF1*-rs34011-AA carriers, 37 patients were heterozygous *FGF1*-rs34011-A/G carriers, and 48 patients were *FGF1*-rs34011-GG carriers. The chi-square test was used to assess whether the allele frequencies agreed with expectation in Hardy-Weinberg equilibrium (HWE). Statistical significance was set at P< 0.05.

### MRI Acquisition, Cortical Volumetric Analysis and Structural Covariance Analysis

MRI images were acquired on a GE 3T Signa Excite scanner (GE Medical System, Milwaukee, WI). The scanning protocol of T1-weighted imaging included inversion-recovery-prepared, three-dimensional, spoiled, gradient-recalled acquisition in a steady-state sequence with a repetition time/inversion time of 8,600 ms/450 ms, 240 × 240 mm field of view, and 1-mm slice thickness.

Statistic Parametric Mapping software version 12 (SPM 12) (www.fil.ion.ucl.ac.uk/spm/software/) was used to pre-process T1 MRI, and was involved to remove non-relevant tissue, for intensity and spatial normalization to the Montreal Neurological Institute space, and for tissue segmentation. Using segmentation in SPM 12, the images were segmented into grey matter and white matter. The regional labeling was identified after aligning to the automatic anatomical label structures and the hippocampal volume (HV) was extracted based on individual segmented GM. The raw HV and total intracranial volume (TIV) were estimated with surface-based atlas maps in Computational Anatomy Toolbox 12 in SPM12 [31].

### Neuropsychological Assessments

EM was assessed using the Chinese Version Verbal Learning Test (CVVLT) [32], by assessing free recall (number of items retrieved over four learning trials of a 9-word list) after 30 seconds (CVVLT-30 s), after 10 minutes (CVVLT-10 min), and cued recall (CVVLT-cued; number of words recalled with cued procedures over four learning trials). CVVLT-30 s and CVVLT-10 min were used to evaluate immediate and delayed recall, and CVVLT-cued was used to measure memory under cue response. The CDR and Mini-Mental State Examination [33, 34] assessed the general intellectual function. Moreover, executive function (Digit Span Backward, Trail Making Test B [35], language (Category Fluency of animal naming [36] and 15-item Boston Naming Test [37]), and visuospatial function (Visual Object and Space Perception Battery [38] and modified Rey-Osterrieth complex figure copy [39]) were also assessed.

### Statistical Analysis

Clinical data and volume in left and right HV were expressed as mean ± standard deviation. The independent t-test with false discovery rate (FDR) correction was used to compare continuous variables among the ε4+ carriers versus ε4- carriers, as well as GG- versus A-allele-carriers. EM performance score and voxel-based morphometry (VBM) were analyzed using two-way analysis of variance (ANOVA) to identify the contribution of interaction effects of *APOE* (ε4+ versus ε4- carriers) with *FGF1* (GG- versus A-allele-carriers). Based on the study rationale, the patients were further classified into four genotypic groups: ε4+ carriers with *FGF1-*rs34011-GG genotype (ε4+/GG-carriers); ε4+ carriers with *FGF1-*rs34011-A-allele genotype (ε4+/A-allele-carriers); ε4- carriers with *FGF1-*rs34011-GG genotype (ε4-/GG-carriers); and ε4- carriers with *FGF1-*rs34011-A-allele genotype (ε4-/A-allele-carriers). Analysis of variance with Bonferroni correction for multiple comparisons was used compare continuous variables among the four genotypic groups.

**Table 1:** Demographic and clinical data of patients with Alzheimer’s disease grouped based on *APOE*-ε4 carriers versus non-carriers or *FGF1*-rs34011-GG (GG-carriers) versus *FGF1*-rs34011-A-allele carriers (A-allele-carriers).

	*APOE*-ε4 carriers	*APOE*-ε4 non-carriers	P value	GG-carriers	A-allele- carriers	P value
Sample size (n)	38	59		48	49	

Age (years)	71.2±7.3	71.7±8.1	0.765	71.1±8.5	71.9±7.0	0.597

Sex (% male)	47.4%	59.3%	0.248	58.3%	51.0%	0.469

Education (years)	8.0±2.3	8.7±4.9	0.502	8.6±4.9	8.2±5.3	0.701

MMSE	21.2±5.7	22.1±6.1	0.449	21.0±6.6	22.5±5.1	0.204

CDR	0.6±0.3	0.5±0.2	0.282	0.58±0.28	0.53±0.24	0.319

Episodic memory scores						

CVVLT-30 s	4.2±2.8	5.1±2.6	0.133	4.6±2.7	4.9±2.7	0.497
CVVLT-10 min	2.7±3.3	4.2±3.1	0.034	3.5±3.3	3.8±3.2	0.567
CVVLT-cued	3.6±3.2	4.9±2.6	0.033	4.3±3.0	4.6±2.9	0.637

TIV (liter)	1.4±0.1	1.4±0.2	0.822	1.3±0.2	1.4±0.1	0.551

TIV adjusted volume *10^-3^						

Left hippocampus	1.0±0.2	1.2±0.2	0.001	1.1±0.2	1.2±0.2	0.356
Right hippocampus	1.1±0.3	1.3±0.2	0.008	1.2±0.3	1.3±0.2	0.106

Data are presented as mean ± standard deviation; P value denotes significant differences between groups on independent t-test for continuous, and χ^2^ test for dichotomous variables. CDR, Clinical Dementia Rating; CVVLT, Chinese version of the Verbal Learning Test (CVVLT-30 s: words recalled after 30 seconds; CVVLT-10 min: words recalled after 10 minutes; CVVLT-cued: words recalled with cued procedures); *APOE*, apolipoprotein E; *FGF1*, fibroblast growth factor 1; MMSE, Mini-Mental State Examination; TIV, total intracranial volume.

**Table 2:** Two-way analysis of variance voxel-based morphometry showing effect of *APOE-FGF1* interactions on structural atrophy in grey matter.

	x	y	z	F-score	Voxels
Right hippocampus	21	-18	-13.5	9.7164	1410
Left hippocampus	-31.5	-15	-12	10.1648	1522
Right inferior temporal gyrus	42	-1.5	-31.5	9.8974	167
Right middle temporal gyrus	48	-48	-1.5	11.4739	203

All significances were set at threshold of uncorrected p< 0.01 at voxel level and false discovery rate corrected p< 0.05 at cluster level. *APOE*, apolipoprotein E; *FGF1*, fibroblast growth factor 1. xyz, local maxima coordinates on Montreal Neurological Institute template brain.

We used two-tailed Spearman’s correlation test to analyze the relationship between bilateral HV and EM scores in each genotypic group. We then used Fisher transformation to further analyze the differences in correlation coefficient value of ρ between each genotypic group measuring the relation of EM performances with HV. All statistical analyses for continuous variables were conducted using SPSS software (SPSS version 22 for Windows®, SPSS Inc., Chicago, IL).

**Table 3:** Correlations between memory performance scores and hippocampal volume.

	All patients with AD	ε4+/GG- carriers	ε4+/A-allele- carriers	ε4-/GG- carriers	ε4-/A-allele-carriers
TIV adjusted left hippocampal volume					
CVVLT-30 s scores	0.525* (<0.001)	0.769* (0.002)	0.454* (0.023)	0.598* (<0.001)	0.230 (0.279)
CVVLT-10 min scores	0.595* (<0.001)	0.812* (0.001)	0.676* (<0.001)	0.533* (0.001)	0.263 (0.215)
CVVLT-cued scores	0.526* (<0.001)	0.518 (0.070)	0.574* 0.003	0.505* (0.002)	0.143 (0.506)
TIV adjusted right hippocampal volume
CVVLT-30 s scores	0.554* (<0.001)	0.837* (<0.001)	0.606* (0.001)	0.493* (0.003)	0.278 (0.189)
CVVLT-10 min scores	0.611* (<0.001)	0.745* (0.003)	0.757* (<0.001)	0.524* (0.001)	0.229 (0.282)
CVVLT-cued scores	0.564* (<0.001)	0.631* (0.021)	0.665* (<0.001)	0.467* (0.005)	0.202 (0.506)

Data are presented as ρ (p value); *p< 0.05; AD, Alzheimer’s disease; CVVLT, Chinese version of the Verbal Learning Test (CVVLT-30 s: words recalled after 30 seconds; CVVLT-10 min: words recalled after 10 minutes; CVVLT-cued: words recalled with cued procedures); ε4+/GG-carriers: apolipoprotein E (*APOE*)-ε4 carriers with fibroblast growth factor 1 (*FGF1*)-rs34011-GG genotype; ε4+/A-allele-carriers: *APOE*-ε4 carriers with *FGF1*-rs34011-A-allele genotype; ε4-/GG-carriers: *APOE*-ε4 non-carriers with *FGF1*-rs34011-GG genotype; ε4-/A-allele-carriers: *APOE*-ε4 non-carriers with *FGF1*-rs34011-A-allele genotype; TIV, total intracranial volume.

## RESULTS

### Clinical and pathological difference between ε4+ carriers and ε4- carriers

We first aimed to characterize the clinical and pathological differences in the ε4+ versus ε4- carriers and GG- versus A-allele-carriers. The distribution of *APOE*-ε4/ε4 carrier genotype conformed to HWE with X^2^= 0.019 (p=0.890), whereas the distribution of *FGF1-*rs34011-AA genotype conformed to HWE with X^2^= 1.288 (p= 0.256). Allele frequencies did not violate the expectation in HWE. Ninety-seven patients with AD completed the study. Their demographic, EM performance and HV are presented in Table 1. There was no significant difference in executive function, language, visuospatial function and TIV between these genotypic groups (P> 0.05).

In independent t-test after FDR correction, the ε4+ carriers had a trend of lower scores in CVVLT-10 min (P= 0.034) and CVVLT-cued (P= 0.033) than the ε4- carriers. In structural study, the ε4+ carriers had a significant smaller left (P= 0.001) and right (P= 0.008) HV than the ε4- carriers after FDR correction (Table 1).

### Clinical and Pathological Changes across Genotypic Groups

**Figure 1.**
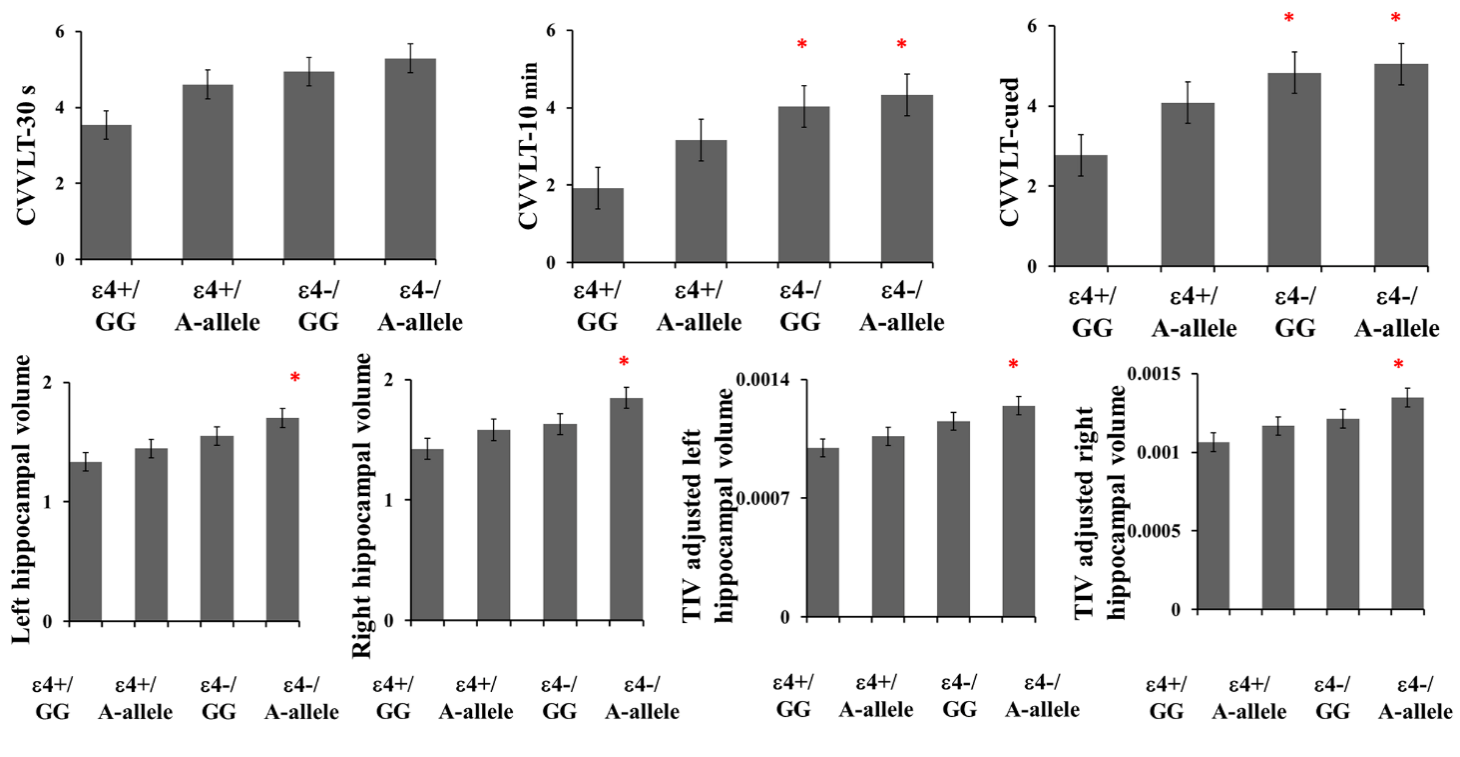
Episodic memory and hippocampal volume among genotypic groups Plot displaying (**A**) scores in episodic memory performance and (**B**) hippocampal volume in each genotypic group. *P< 0.05 as compared with the ε4+/GG group. CVVLT, Chinese version of the Verbal Learning Test (CVVLT-30 s: words recalled after 30 seconds; CVVLT-10 min: words recalled after 10 minutes; CVVLT-cued: words recalled with cued procedures); ε4+/GG: *APOE*-ε4 carriers with *FGF1*-rs34011-GG genotype; ε4+/A-allele: *APOE*-ε4 carriers with *FGF1*-rs34011-A-allele genotype; ε4-/GG: *APOE*-ε4 non-carriers with *FGF1*-rs34011-GG genotype; ε4-/A-allele: *APOE*-ε4 non-carriers with *FGF1*-rs34011-A-allele genotype; TIV, total intracranial volume.

To investigate how the *FGF1* (rs34011) genotype modified the detrimental effect of the *APOE*-ε4 carrier genotype on EM and the HV, we categorized the patients into ε4+/GG-carriers, ε4+/A-allele-carriers, ε4-/GG-carriers and ε4-/A-allele-carriers and compared EM performance and HV among the genotypic groups.

There was no significant difference in age, educational level, and CDR among the four genotypic groups (P> 0.05). Among the four genotypic groups (Fig. 1), dose-dependent gradients were observed in delayed retrieval and cued recall, implying possible interaction effects of *APOE* with *FGF1* (rs34011) on EM deficits. In independent t-test, ε4-/A-allele-carriers and ε4-/GG-carriers differed with ε4+/GG-carriers in CVVLT-10 min and CVVLT-cued (P< 0.05) (Fig. 1). There was a significant difference in bilateral HV among the four genotypic groups (Fig. 1). In post-hoc analysis, the ε4-/A-allele-carriers had a significantly larger bilateral HV than the ε4+/GG-carriers (P< 0.05) (Fig. 1), and the ε4-/A-allele-carriers also had significantly larger left HV than the ε4+/A-allele-carriers (P= 0.024).

### FGF1 Genotype Modulated the EM Impairment and Structural Atrophy in ε4+ carriers

We then further analyzed the interaction effect of *APOE* with *FGF1* on EM deficits and structural changes using VBM.

**Figure 2.**
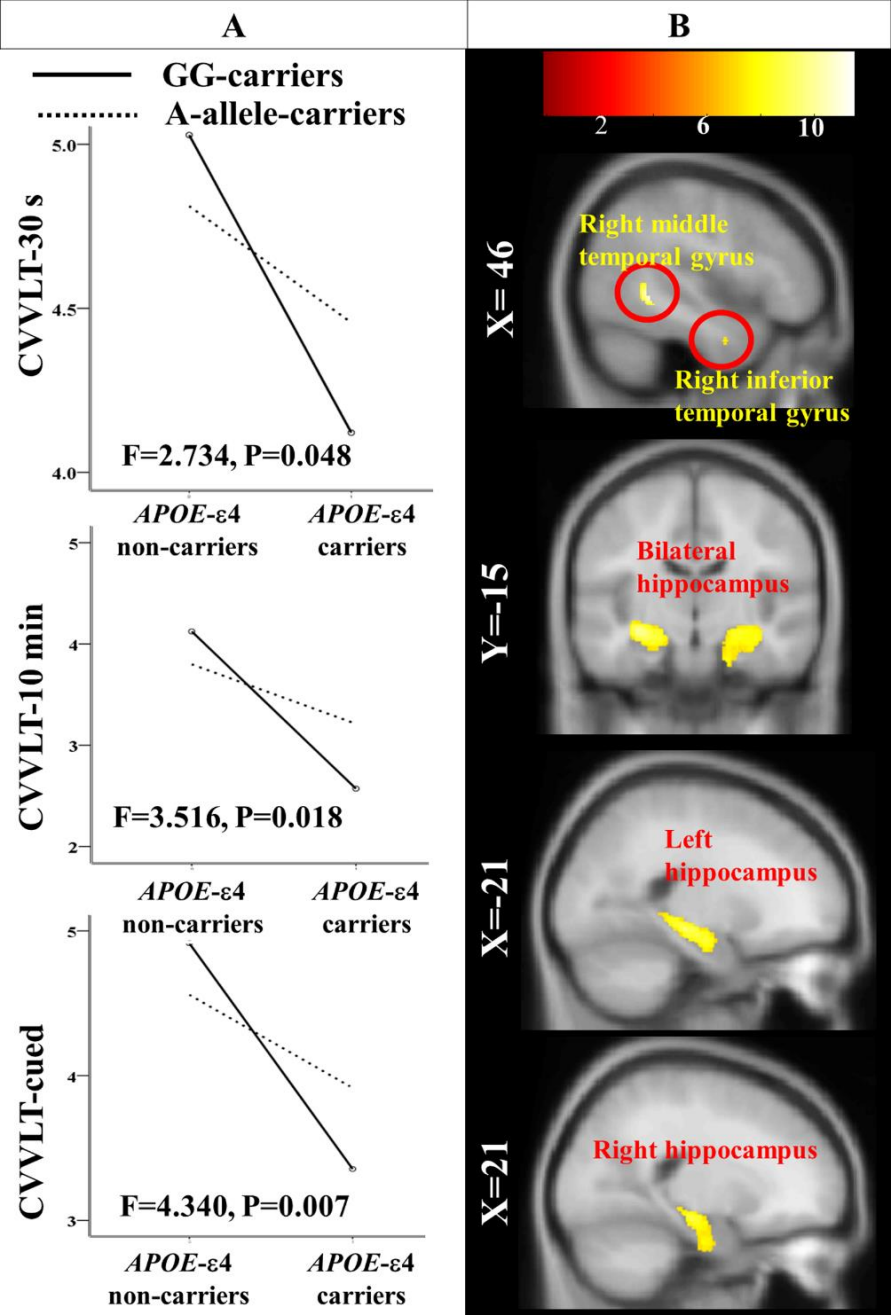
Genetic interaction effects on episodic memory and regional volume (**A**) Effect of *APOE-FGF1* (rs34011) interaction on scores in episodic memory performance; (**B**) Statistical maps of *APOE-FGF1* (rs34011) interaction effect on regional atrophy on Montreal Neurological Institute template brain. A-allele-carriers: *FGF1*-rs34011-A-allele carriers; CVVLT, Chinese version of the Verbal Learning Test (CVVLT-30 s: words recalled after 30 seconds; CVVLT-10 min: words recalled after 10 minutes; CVVLT-cued: words recalled with cued procedures); GG-carriers: *FGF1*-rs34011-GG carriers.

After controlling for disease severity, there were interaction effects of *APOE* with *FGF1* on deficits in CVVLT-30 s (F4,92= 2.734, p= 0.048), CVVLT-10 min (F4,92= 3.516, p= 0.018) and CVVLT-cued (F4,92= 4.340, p= 0.007) (Fig. 2A).

In two-way ANOVA VBM analysis, after controlling for disease severity, there was a significant interaction effect of *APOE* with *FGF1* (rs34011) on regional atrophy in right inferior and middle temporal gyrus, right hippocampus, left hippocampus (p< 0.01) (Fig. 2B; Table 2).

### Different Relationship between EM and HV among Genotypic Groups

To investigate the genotypic effect on clinical-pathological relationship, we separately analyzed the relationship between HV and EM performance within each genotypic group, separately (Table 3).

Among all of the enrolled patients with AD, the scores in CVVLT-30 s, CVVLT-10 min and CVVLT-cued were correlated with bilateral HV (p<0.05) (Table 3).

In analysis of individual genotypic group, the scores in CVVLT-30 s, CVVLT-10 min and CVVLT-cued were correlated with bilateral HV in all of the groups (p< 0.05) except for the ε4-/A-allele-carriers (p> 0.05; Table 2).

We then further analyzed the differences in correlation coefficient value of ρ between each genotypic group measuring the relation of EM impairment with HV. Comparison using Fisher transformation showed that significant difference in ρ value measuring the relation of CVVLT-30 s and CVVLT-10 min with left HV, and on the relation of CVVLT-30 s with right HV between ε4+/GG-carriers and ε4-/A-allele-carriers (P< 0.05).

## DISCUSSION

### Main Findings

There are three major findings. First, among the four genotypic groups, dose-dependent gradients were observed in bilateral HV, implying a possible effect of *APOE*-*FGF1* (rs34011) interaction on HA. Additionally, there was an interaction effect of *APOE* with *FGF1* (rs34011) on bilateral hippocampus in VBM. Second, there was an interaction effect of *APOE* with *FGF1* (rs34011) on EM deficits. Third, we demonstrated a genotypic effect on the association between HA and EM deficits. No significant relationship between EM performance and HV was shown in ε4-/A-allele-carriers, whereas HV was positively correlated with EM function scores in the other three genotypic groups.

### Interaction Effects of APOE with FGF1 (rs34011) on the Hippocampus

The apoE exerts protective mechanisms via maintaining neuronal integrity and regeneration process in neurodegeneration-susceptible regions [25], such as the hippocampus. One previous study indicates that the *APOE*-ε4 carriers would have greater HA than *APOE*-ε4 non-carriers [7]. Therefore, the protective mechanisms of apoE may be reduced by the *APOE*-ε4 carrier genotype [26, 27]. To rescue neurodegeneration-associated neuronal and synaptic dysfunction, *FGF1* (rs34011) may show functional significance though promoting survival of neurons, suppressing neurotoxicity, preventing Aβ spreading, and increasing invasive ability of fibroblast, which may subsequently be converted to functional neurons [17, 18, 20, 40]. Association studies have examined single gene cognitive effects, but fail to produce replicable results [22, 23, 41]. In this study, we demonstrated a possible synergistic adverse effect of the *APOE*-ε4 carrier and *FGF1*-rs34011-GG genotypes on HV, which appeared to decline along a gradient from the ε4-/A-allele-carriers to ε4+/GG-carriers. Moreover, we showed the difference in HV among different genotypic group using strict post-hoc analysis with ANOVA. As dose-dependent gradients in bilateral HV implied possible interaction effects of *APOE* with *FGF1* (rs34011) on HA, VBM-based analysis further showed an effect of *APOE*-*FGF1* (rs34011) interactions on bilateral hippocampus. These results suggested that both *APOE*-ε4 carrier and *FGF1*-rs34011-GG genotypes exerted synergistic and interactive detrimental effect on HV.

### Interaction Effects of APOE with FGF1 (rs34011) on EM Deficits

Typical AD begins with EM deficits characterized by encoding and recall [42]. The typical amnestic clinical syndrome has been associated with HA [10]. Although the *APOE*-ε4 carrier genotype has been shown to have detrimental effect on HV [7, 43], ε4+ carriers have been shown to exhibit inconsistent associations with EM impairment [8, 14, 15]. In this study, we investigated whether genetic variations in the *APOE* and *FGF1* (rs34011) could partially explain the inconsistent heritability of the detrimental effect of the *APOE*-ε4 carrier genotype on EM deficits in AD.

To the best of our knowledge, this is the first study to report the interaction effects of *APOE* with *FGF1* on EM impairment in a cohort comprised of subjects with mild AD [14]. The interaction was possibly through an *FGF1* (rs34011)-dependent effect exerted by variations in the *APOE*-ε4 carrier status. The detrimental effects of the *APOE*-ε4 carrier genotype on EM function were more pronounced in the GG-carriers than in the A-allele-carriers.

In spite of an effect of *APOE*-*FGF1* interactions on EM impairment, we only found a trend of difference in EM performance between the ε4+ and ε4- carriers, and among different genotypic groups, using strict post-hoc analysis. This observation was generally in agreement with previous negative findings [7, 8]. Although strict post-hoc analysis did not show significant differences in EM performance among different genotypic groups, dose-dependent gradients were observed. Using independent t-test, we showed that ε4+/GG-carriers had significant lower EM performance than ε4-/GG-carriers and ε4-/A-allele-carriers. It suggested a possible synergistic detrimental effect of the *APOE*-ε4 carrier and *FGF1*-rs34011-GG genotypes on EM performance.

No significant difference between ε4-/GG-carriers and ε4+/A-allele-carriers may be helpful in explaining the missing heritability of the detrimental effect of the *APOE*-ε4 carrier genotype on EM deficits in some patients with AD [8].

### The Relationship between EM Performance and HV

There was a significant association between HA and EM deficits in three of the four genotypic groups, including ε4+/GG-, ε4+/A-allele-, and ε4-/GG-carriers. This relationship was strongly supported by existing literature about the hippocampus-associated EM impairment in AD [10, 44]. This clinical-pathological relationship in patients with AD is more pronounced than that in cognitively normal subjects [10, 45,46]. The lack of relation of HV with EM performance has been attributed to insufficient variability in HV in cognitively normal subjects.

In the current study, we showed that the EM performance was not associated with HV in ε4-/A-allele-carriers. The clinical-pathological relationship in this genotypic group was different from that in other three genotypic groups. It suggested that genetic basis may affect the relation of EM performance with HV.

The lack of association between HV and EM performance within ε4-/A-allele-carriers with AD might be attributed to the restrictive variability in HV in this genotypic group, similar to cognitively normal subjects [10, 45, 46]. The observation suggests the synergistic protective effects of *APOE*-ε4 non-carrier and *FGF1*-rs34011-A-allele genotypes on HA. However, as ε4-/A-allele-carriers did not show significant better EM function than other genotypic groups, according to the strict post-hoc analysis, the genotypic protective effects remained controversial on EM function preservation.

Cholinesterase inhibitors (ChEIs) are among the sole treatments available for AD. Owing to their cholinergic effects on hippocampus, ChEIs play a critical role in hippocampus-dependent memory performance [47, 48]. As therapeutic effect of ChEIs may be associated with hippocampal pathogenesis, the lack of relation of HA with EM deficits in ε4-/A-allele-carriers suggests that multiple interactions among different genetic-biological systems may influence several aspects of disease presentation and therapeutic effect. Clarifying genotype-associated pattern of clinical features and treatment efficacy in AD may be useful for identifying high risk or responder individuals.

Conclusively, our results suggest genotype-related variation in the relationship between EM deficits and HA. Moreover, the ε4-/A-allele-carriers may harbor protective effect on vulnerable neurons.

### Limitations

There were three limitations. First, as complex interactions among multiple SNPs within susceptibility genes have been identified in sporadic AD, the effects of gene-gene interactions on hippocampus owing to merely two different susceptibility genes might be unable to fully explain the pathologic changes in AD. Further study is needed to explore the complicate genotypic effect on AD pathogenesis. Second limitation was the small sample size. However, we used strict post-hoc analysis with ANOVA to investigate the variation in HV and EM performance among different genotypic groups to avoid statistical errors, and we made a careful interpretation with regards to the differences in EM impairment among the genotypic groups. Moreover, the strength and consistency of our results lied in that both volume-of-interest and VBM analyses suggested interaction effects of *APOE* with *FGF1* (rs34011) on HA. Third limitation was lack of normal controls in this study. Nonetheless, we aimed to explore the genotypic effect on heterogeneity of clinical-pathological relationship in AD, which might be useful to investigate the genotypic effect on therapeutic efficacy. Longitudinal follow-up will be needed to further investigate the role of genotype-associated variation in clinical and pathological progression of AD, and the genotypic effects on clinical-pathological relationship in patients with moderate to severe AD in addition to those with mild AD. Further studies include the pathological effect of neuritic plaque and neurofibrillary tangles on genotype-associated clinical variation will be helpful for fully understanding the pathogenic mechanism in AD.

## Conclusions

In conclusion, we identified an interaction effect of *APOE* and *FGF1* (rs34011) on HV and EM function. There was genotypic effect on clinical-pathological relationship in AD. Clarifying genotype-associated pathophysiology of AD might be useful to identify high risk or responder individuals in the treatment for AD.
